# Supplementary material for: Angiotensin I-Converting Enzyme (ACE) Inhibition and Molecular Docking Study of Meroterpenoids Isolated from Brown Alga, Sargassum macrocarpum
Source: Int J Mol Sci. 2023 Jul 4;24(13):11065. doi: 10.3390/ijms241311065 (PMC10341620; doi:10.3390/ijms241311065)
Supplement: Supplementary file 1 [file ijms-24-11065-s001.zip › ijms-2436836-supplementary.pdf]

Supporting information

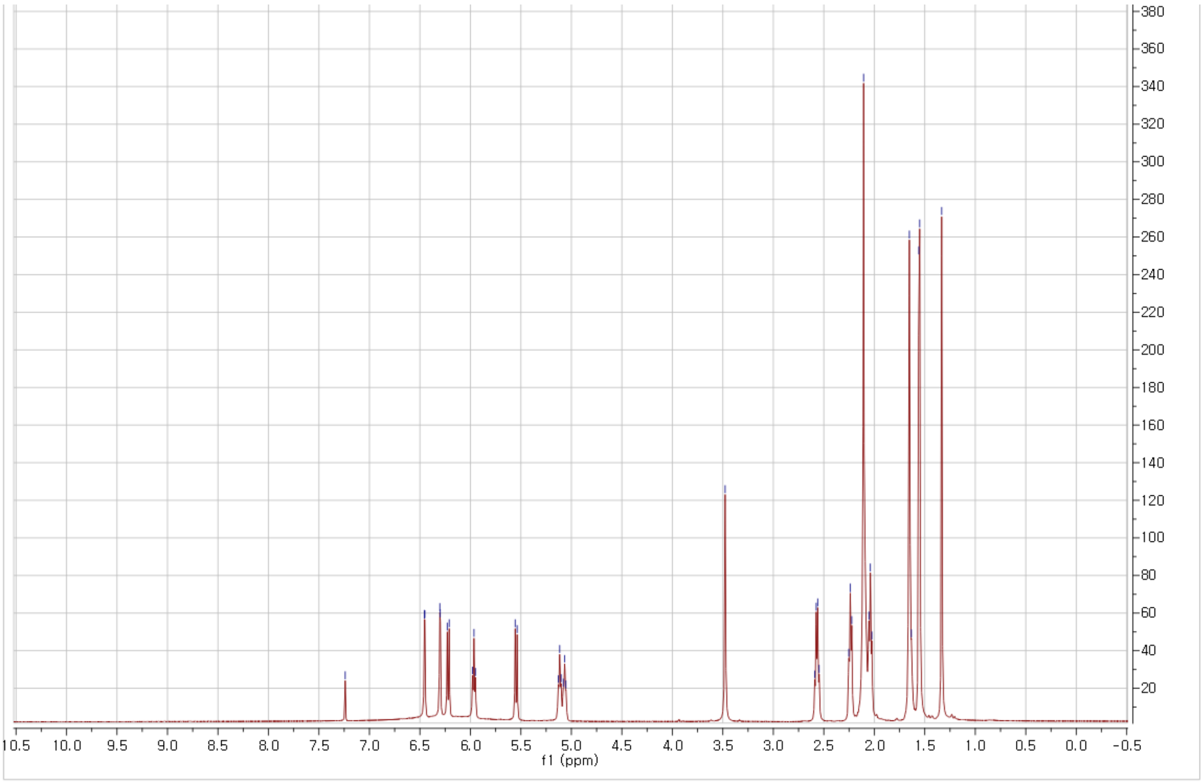

Figure S1. ^{1}H NMR spectrum of sargachromenol (**1**)

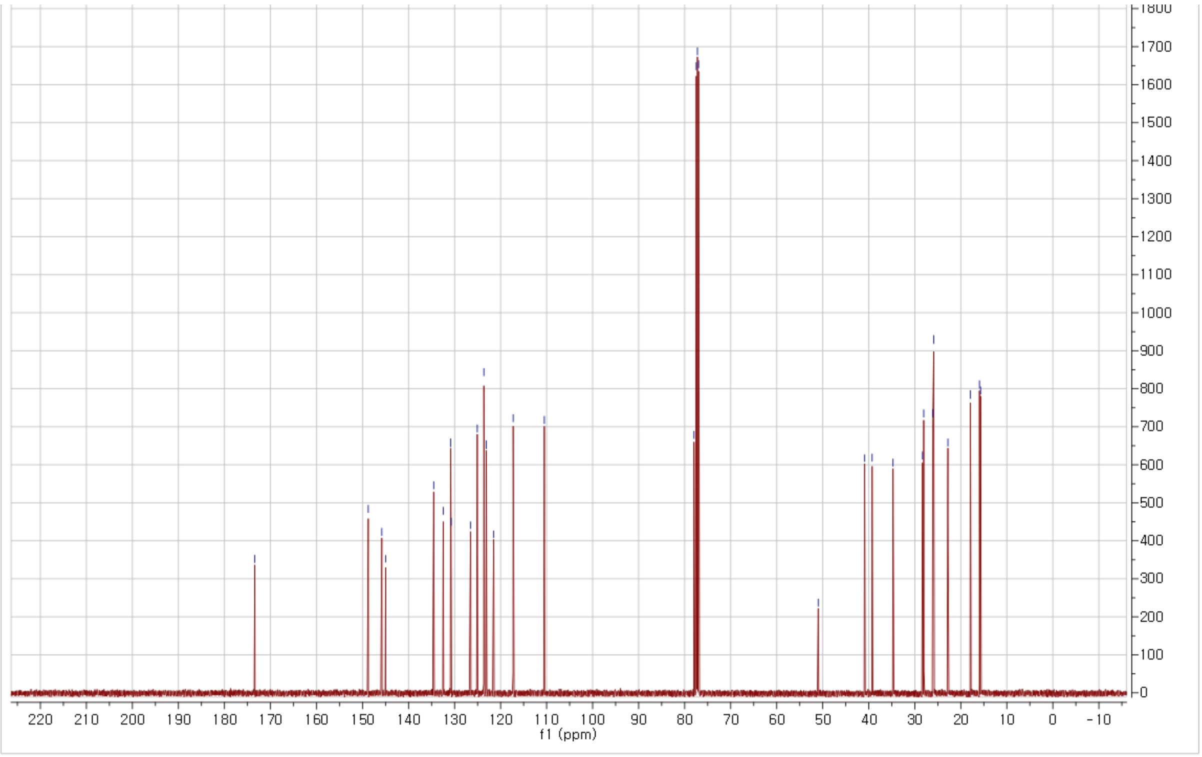

Figure S2. ^{13}C NMR spectrum of sargachromenol (**1**)

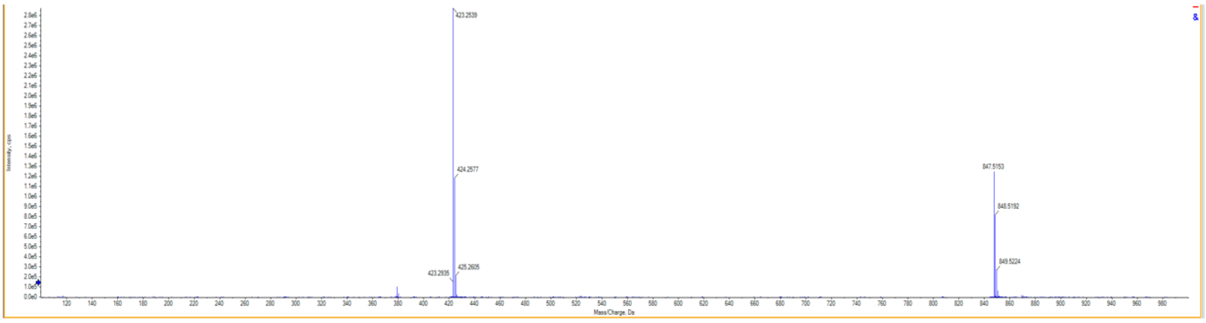

Figure S3. HR-ESI-MS spectrum of sargachromenol (**1**)

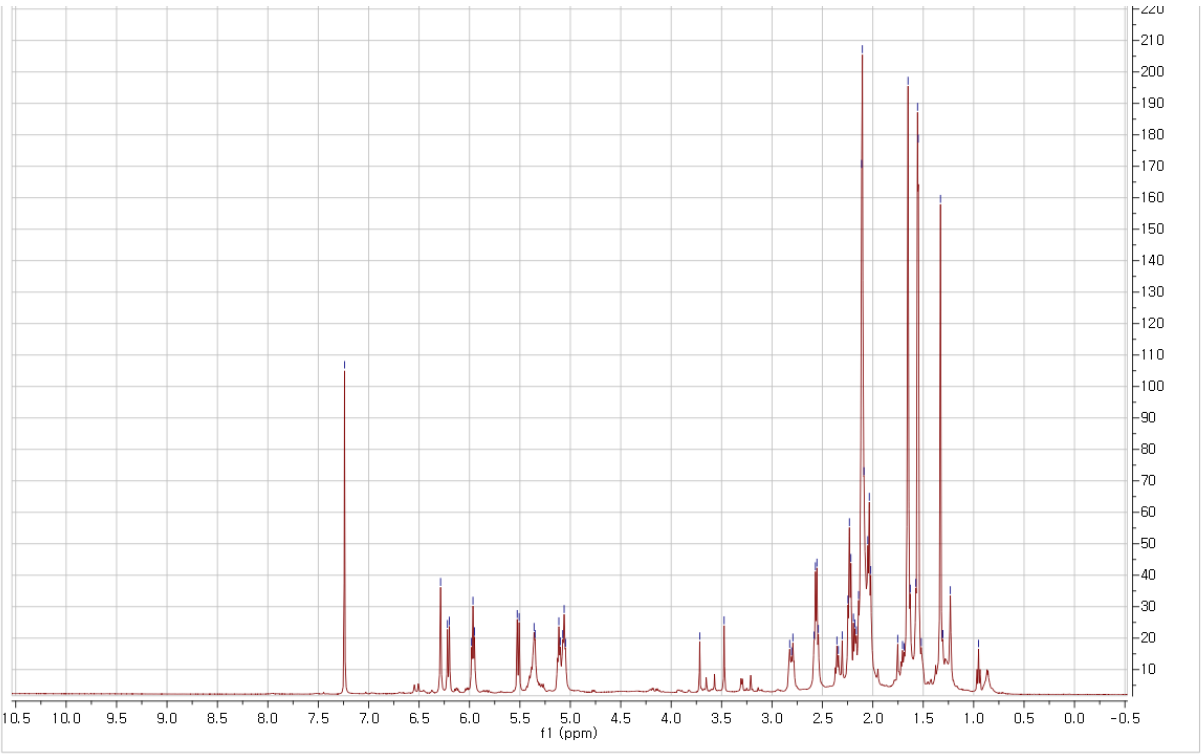

Figure S4. ^{1}H NMR spectrum of 7-methyl sargachromenol (**2**)

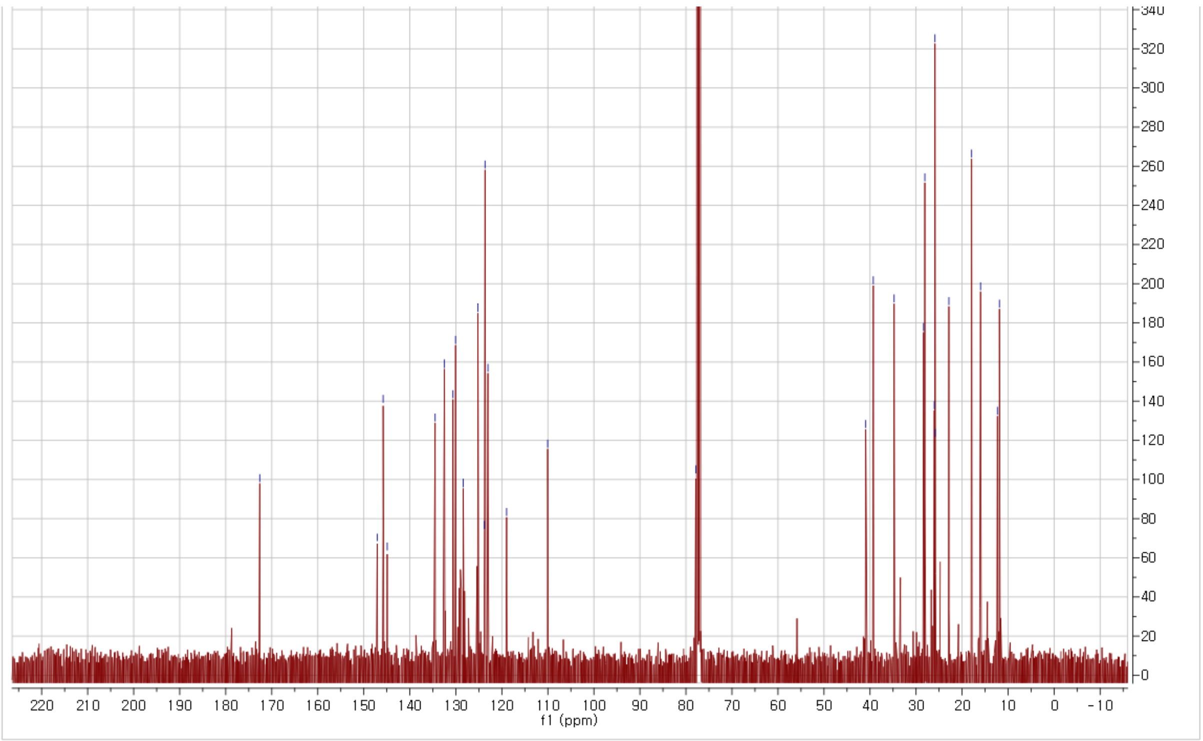

Figure S5. ^{13}C NMR spectrum of 7-methyl sargachromenol (**2**)

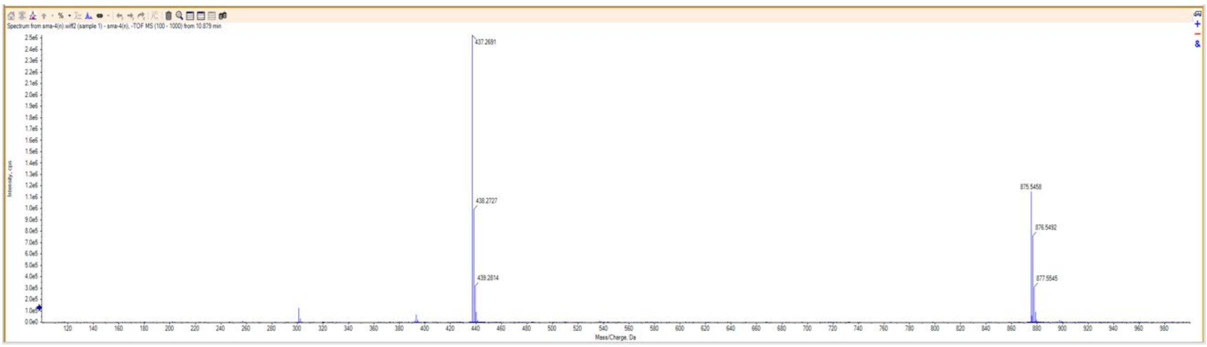

Figure S6. HR-ESI-MS spectrum of 7-methyl sargachromenol (**2**)

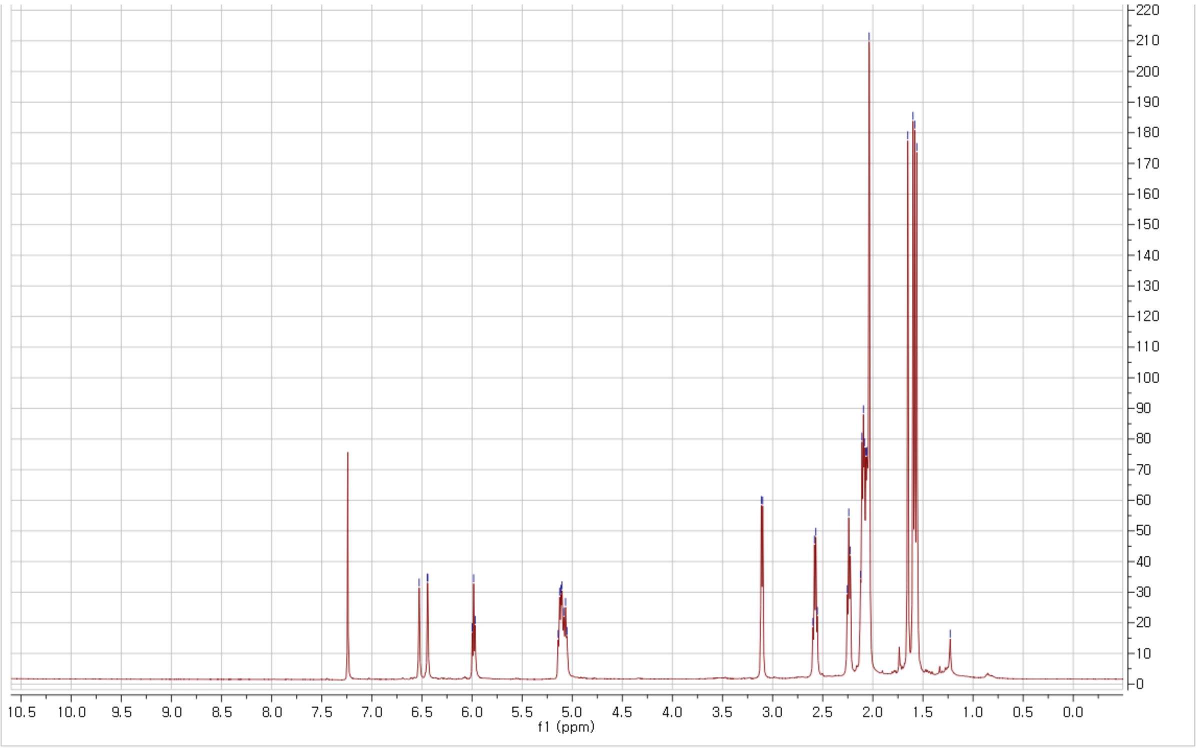

Figure S7. ^{1}H NMR spectrum of sargaquinoic acid (**3**)

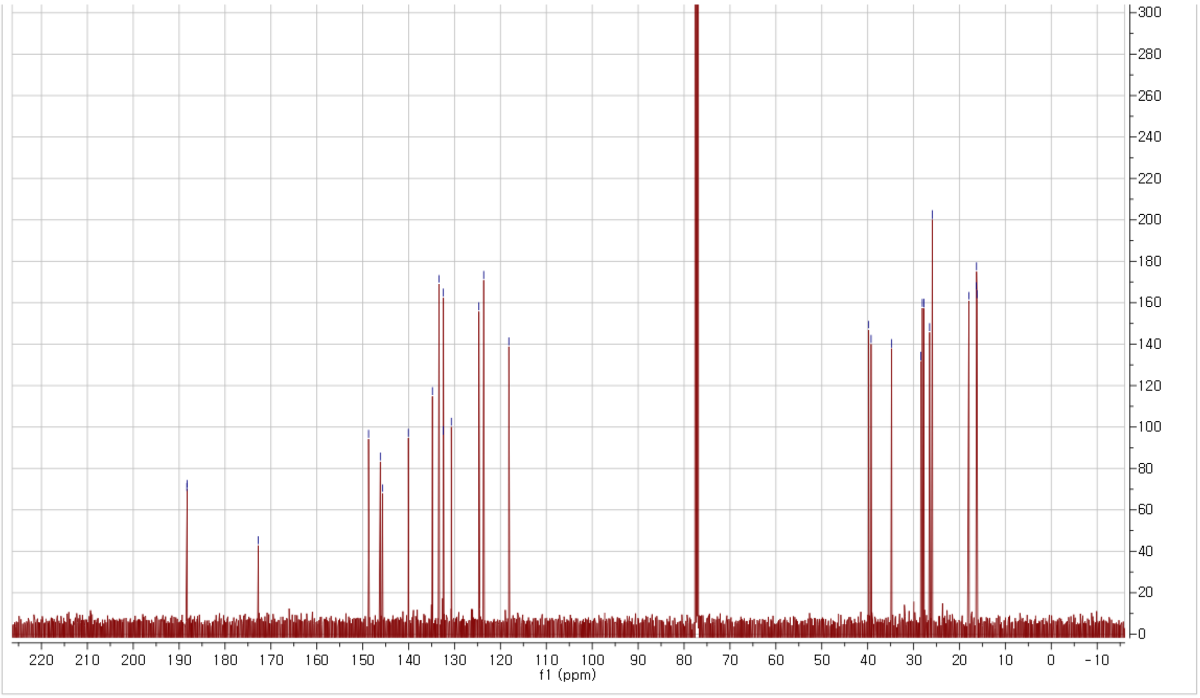

Figure S8. ^{13}C NMR spectrum of sargaquinoic acid (**3**)

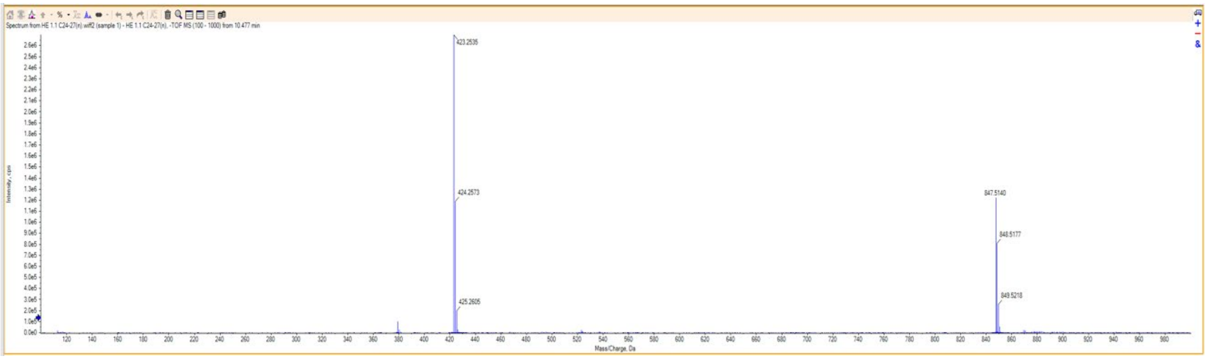

Figure S9. HR-ESI-MS spectrum of sargaquinoic acid (**3**)
